# 

**Published:** 1856-09

**Authors:** 


### CONTENTS.

### ORIGINAL COMMUNICATIONS.

PAGE

ARTICLE I.—Dry Gangrene. By CHARLES WILLIAM ASHBY, M.D., Alexandria, Virginia, ... 1

ARTICLE II.—Case of Catalepsy, with Remarks. By H. W. CAFFEY, M.D., Collirene, Alabama, ... 3

ARTICLE III.—Observations upon Menstruation—its Cause, Character and Effects upon the Female Economy. By ROBERT E. CAMPBELL, M. D., Benton, Lowndes County, Alabama, ... 10

ARTICLE IV.—Report of a Case of Gun-shot Wound, with observations. By WALTER SOMERVILLE, M. D., Culpepper County, Virginia, ... 15

ARTICLE V.—Gaseous Colic. By W. T. GRANT, M. D., of Wrightsboro', Columbia County, Georgia, ... 17

### SELECTIONS.

Congenital Hydrocephalus, ... 19

Female Education, ... 26

Of the possible cure of Suppurative Arthritis, with the preservation of mobility, 31

On the Use and Abuse of Chemical Baths, ... 37

Homœopathy, ... 40

The Effects of Dentition on Nursing Children, ... 43

Case of Congenital Cephalocele, ... 47

Æsthetics of Medicine, ... 50

### EDITORIAL AND MISCELLANEOUS.

Close of Lectures, ... 52

Reports and Announcements Received, ... 54

New Orleans School of Medicine, ... 55

Pennsylvania College of Medicine, ... 5

Number of Matriculates in the Atlanta Medical College, ... 55

Appointment of L. J. Robert, M.D., to the Chair of Physiology and Pathology of the Oglethorpe Medical College at Savannah, Georgia, ... 56

Record of Medical Science, ... 56

